# Editorial: Antimicrobial Usage in Companion and Food Animals: Methods, Surveys and Relationships With Antimicrobial Resistance in Animals and Humans

**DOI:** 10.3389/fvets.2020.00063

**Published:** 2020-02-12

**Authors:** Miguel A. Moreno, Lucie Collineau, Carolee A. Carson

**Affiliations:** ^1^Department of Animal Health, Veterinary Faculty, Complutense University, Madrid, Spain; ^2^VISAVET Health Surveillance Centre, Complutense University, Madrid, Spain; ^3^Epidemiology of Animal and Zoonotic Diseases Unit, French National Institute for Agricultural Research (INRA), Marcy l'Etoile, France; ^4^Public Health Agency of Canada (PHAC), Guelph, ON, Canada

**Keywords:** antibiotic use, metrics, livestock, pets, monitoring, surveillance

The best way to quantify antimicrobial use (AMU) in animals is still an elusive question, it probably does not have a unique answer. This collection of 15 articles describes different metrics, methodologies, data sources, animal scenarios, study designs, and levels of study about AMU quantification in animals. The diversity of approaches highlights a strong need for international collaboration, sharing of experiences, and more discussion about methods to improve uptake of harmonized standards (where harmonization might be suitable).

The less controversial aspect of this topic is that there was consensus among these articles that a relative measure is needed, dividing amounts of antimicrobials (numerator) by a denominator summarizing the animal population at risk of being treated. However, both the numerator and denominator have their specific challenges. In addition, a period of time for data collection must be fixed or considered. In the human arena, a standardized population approach based on established defined daily doses (DDDs) and census data is utilized around the world, delivering information on the number of DDDs per population and year (or days) (1). Nevertheless, mimicking this procedure in animals is not an easy subject, with many controversial facets previously described (2).

The use of weight-based (e.g., mg or kg of active ingredient) vs. dose-based (e.g., DDD) metrics in the numerator was discussed in several papers of this collection. A main advantage of weight-based metrics is their higher availability (i.e., the data comprising these metrics are more often available), that make them a more accessible option for worldwide AMU monitoring (Góchez et al.). However, Brault et al. demonstrated that dose-based metrics were more accurate than weight-based metrics when there was variation in the type (e.g., concentrations and durations of effect) of antimicrobials used by the populations being compared. The studies by Agunos et al., Brault et al., and Van Cuong et al., where weight-based and dose-based metrics were applied to the same AMU data, all demonstrated a significant impact of those metrics on the study results, that could even lead to different conclusions (e.g., increase vs. decrease in AMU over time in turkeys in Agunos et al.). Agunos et al. stressed the added value of using multiple AMU indicators for monitoring the impact of stewardship activities and interventions. Nonetheless, weight-based and dose-based metrics are not mutually exclusive, and it is possible to convert one into another (Stebler et al.).

Two articles in this collection addressed defining or establishing national “animal” or “vet” (a linguistic discussion not yet resolved) DDDs for pigs (Bosman et al. in Canada; Echtermann et al. in Switzerland) and poultry (Bosman et al. in Canada), as a tool for the calculation of the number of DDDs per animal population and year (a proxy of the number of treatments-day), at the country or region-level. This indicator is also discussed and used in the article of Brault et al. In addition, national vet defined course doses (DCDs) for pigs have been proposed in Switzerland in the article of Echtermann et al. to calculate the number of DCDs per population and year (a proxy of the number of treated animals). A similar exercise for calculation of the number of treated animals in pigs and calves in Switzerland is presented in the article of Stebler et al. National DDDs lists proposed by Bosman et al. and DDDs and DCDs lists proposed by Echtermann et al. differed from those proposed by the European Medicines Agency for certain antimicrobial classes (3), reflecting the need for individual countries to develop their own lists for more precise AMU quantification at the national level, while the EMA lists may be preferred for international comparisons.

Both the number of DDDs and the number of DCDs are indicators based on standardized measurements that do not necessarily reflect the real or actual AMU. For a more real AMU estimation in a given population with detailed data available, the used daily dose (UDD) may be a better choice to reflect what is happening in that specific population in terms of selection pressure. This is explored in the articles of Kasabova et al. (pigs and broilers), Brault et al. (beef feedlot), and Waret-Szkuta et al. (pigs). All three papers highlighted that the choice of DDD vs. UDD had an impact on the results. Interestingly, Kasabova et al. recommended using UDD-based calculations to run monitoring systems with a benchmark mission. Should DDD be preferred to compare AMU between populations, additional considerations should be made to adjust for discrepancies between DDD and UDD.

The third parameter having a huge effect on AMU indicators is the animal weight. The article of Brault et al. addressed this question in beef cattle, where the use of estimated vs. actual weights notably influenced the results obtained. Similar observations were made in pigs by Waret-Szkuta et al. Equally, the use of weight at treatment vs. the weight at slaughter (Góchez et al.) or the weight sold (Van Cuong et al.) had a strong impact on calculations, especially for larger livestock species like cattle and pig.

Sales of veterinary medical products containing antimicrobials are a classical source of raw data for AMU consumption calculations (Góchez et al.; Stebler et al.). Nevertheless, prescriptions because they have more detailed information closer to the end-users, may be a more accurate source of possible selection pressure; prescriptions were used in the articles of Hommerich et al. and Hopman et al. to calculate AMU in German cattle and Dutch companion animals, respectively.

Although most of the literature on AMU in this collection focused on pigs and cattle (half of the articles of this Research Topic), four articles considered AMU in poultry (Kasabova et al.; Van Cuong et al.; Agunos et al.; Bosman et al.), all of them using the above mentioned DDD approach. Data on AMU from pets were presented in three articles (Singleton et al.; Gómez-Poveda and Moreno; Hopman et al.), using different approaches. Hopman et al. used DDD per clinic and year, whereas Singleton et al. and Gómez-Poveda and Moreno focused on the percentages of prescriptions. Specific scenarios regarding indications for AMU, such as bovine respiratory disease (Brault et al.) and canine acute diarrhea (Singleton et al.) were also presented in this collection, as well as an article exploring drivers for AMU in the pig sector presented by Coyne et al..

Finally, the OIE approach for worldwide AMU monitoring was described in the article of Góchez et al. The OIE view and efforts on this topic are of paramount importance for understanding the different situations around the world where the data may be obtained, and the compromise for a global harmonized methodology to report quantitative data.

In summary, several articles of this collection highlighted that real use data (regarding dose, treatment length and body weight at treatment) were the ideal data for calculating and reporting AMU. Nevertheless, all these data are rarely available simultaneously, hence standard values are the pragmatic alternate choice for calculations. Consequently, transparency about the methods and data used to calculate AMU indicators is needed (Table 1). This was stressed by all the authors in this collection as a pre-requisite to preserve accuracy and understanding of the data, especially when data comparisons are performed.

**Table 1:** This table includes a list of options noted in the research collection for consideration based on available data or objectives of AMU reporting.

	**Basic data**	**Examples**
AMU (numerator)	Data source	Sales, prescriptions, invoices, farm records, others
	Level of measurement	Individual animal, batch/flock/pen, farm, region, country, others
	Timing coverage	Year, production cycle, others
	Dose	Standard (SPC), used, others
	Treatment length	Standard (SPC), used, others
	Index	DDD, UDD, DCD, UCD, mg, kg, others
Population (denominator)	Data source	Farm records, national data bases, census information, FAOSTAT, others
	Level of measurement	Individual animal, batch/flock/pen, farm, region, country, others
	Timing coverage	Year, production cycle, others
	Body weight level	At treatment, at slaughter, at sale, others
	Body weight	Standard (e.g., average weight at treatment), measured, others
	Index	Biomass, population correction unit, number of animals, number of animal-time, others
Indicator	Denomination	Mg of active substance/biomass, Number of DDDs per (10x) animal-time at risk, number of UCD per (10x) animal at risk, others

*SPC, summary of product characteristics; DDD, defined daily dose; UDD, used daily dose; DCD, defined course dose; UCD, used course dose*.

## Author Contributions

MM produced the first draft of the editorial. All authors edited and approved the editorial.

### Conflict of Interest

The authors declare that the research was conducted in the absence of any commercial or financial relationships that could be construed as a potential conflict of interest. The reviewer JD declared a past co-authorship with one of the author LC to the handling editor.
